# 单细胞组学在套细胞淋巴瘤中的应用进展

**DOI:** 10.3760/cma.j.cn121090-20260317-00135

**Published:** 2026-07

**Authors:** 畅 王, 青 张, 鑫 曹

**Affiliations:** 1 福建医科大学肿瘤临床医学院，福建省肿瘤医院淋巴瘤科，福州 350000 Department of Lymphoma, Clinical Oncology School of Fujian Medical University, Fujian Cancer Hospital, Fuzhou 350000, China; 2 南通大学附属医院病理科，南通 226001 Department of Pathology, Affiliated Hospital of Nantong University, Nantong 226001, China; 3 南通大学附属医院血液科，南通 226001 Department of Hematology, Affiliated Hospital of Nantong University, Nantong 226001, China

## Abstract

套细胞淋巴瘤（MCL）是一种具有显著生物学与临床异质性的成熟B细胞肿瘤。近年来，布鲁顿酪氨酸激酶抑制剂、嵌合抗原受体T细胞治疗等改善了部分患者预后，但原发耐药、早期复发及治疗后进展仍是临床管理中的难点。传统检测方法难以充分解析MCL的亚克隆结构、细胞状态转换及肿瘤微环境重塑。单细胞组学及其衍生的空间分辨技术的发展，使研究者能够在细胞和空间维度上分析MCL的克隆演化、免疫生态和耐药相关程序。本文围绕单细胞RNA测序、单细胞B/T细胞受体测序、单细胞DNA测序、单细胞表观遗传学、多模态整合、空间转录组和多重成像技术，综述其在MCL肿瘤内异质性、免疫微环境、治疗耐药机制及临床转化中的研究进展及挑战。

## Abstract (en)

Mantle cell lymphoma（MCL）is a mature B-cell neoplasm characterized by marked biological and clinical heterogeneity. Although novel strategies such as Bruton's tyrosine kinase inhibitors and chimeric antigen receptor T-cell therapy have improved outcomes for a subset of patients, primary resistance, early relapse, and post-treatment progression remain major challenges in clinical management. Conventional bulk-level assays have limited capacity to resolve subclonal architecture, cellular state transitions, and microenvironmental remodeling of MCL. The development of single-cell omics and its derived spatially resolved technologies now enables the profiling of clonal evolution, immune ecology, and resistance-associated programs of MCL at both cellular and spatial dimensions. This review summarizes recent advances in the application of single-cell RNA sequencing, single-cell B/T-cell receptor sequencing, single-cell DNA sequencing, single-cell epigenomics, multimodal profiling, and spatial transcriptomic/imaging approaches to MCL. We focus on their contributions to the understanding of intratumoral heterogeneity, immune microenvironmental remodeling, therapeutic resistance, and potential clinical translation, as well as current challenges.

套细胞淋巴瘤（MCL）占非霍奇金淋巴瘤的6％～8％，其标志性分子事件为t（11;14）（q13;q32）易位导致的细胞周期蛋白D1（CCND1）基因过表达，常伴共济失调-毛细血管扩张突变（ATM）、肿瘤蛋白p53（TP53）、Notch受体1（NOTCH1）基因等遗传学异常[1]–[2]。近年来，布鲁顿酪氨酸激酶抑制剂（BTKi）、B细胞淋巴瘤2蛋白（BCL2）抑制剂及嵌合抗原受体T（CAR-T）细胞治疗等新策略改善了部分MCL患者的预后，但耐药与复发仍较常见[2]–[3]。不同患者在临床缓解深度、缓解持续时间和耐药模式方面存在明显差异[4]–[5]，表明MCL并非单一生物学实体，而是由不同遗传背景、细胞状态和微环境共同塑造的异质性疾病。传统整体水平测序虽能够识别总体突变谱和转录改变，却难以区分亚克隆组成、细胞状态及其与肿瘤微环境的动态互作。随着单细胞组学的发展及相关空间分辨技术的引入，研究者得以在单细胞乃至原位组织分辨率下分析肿瘤细胞状态及其与微环境的相互作用，为理解MCL的生物学异质性提供了新的研究视角。本文旨在总结单细胞组学在MCL肿瘤内异质性、免疫微环境重塑、治疗耐药机制、临床转化方面研究中的主要应用进展，并讨论其潜在的临床意义与当前局限。

一、单细胞组学及相关空间分辨技术在MCL研究中的应用

1. 单细胞RNA测序（scRNA-seq）：scRNA-seq是发展较早且目前应用最为广泛的单细胞组学技术，可进行细胞组成、细胞转录状态及其与微环境之间关系的分析[6]。在MCL中，scRNA-seq不仅有助于识别处于不同增殖、应激或炎症相关程序的恶性B细胞状态，还可呈现T细胞、自然杀伤细胞（NK细胞）、单核或巨噬细胞等免疫群体的组成变化[7]–[9]。但需注意不同部位的样本（淋巴结、骨髓或外周血）在细胞组成及背景方面存在明显差异，组织解离过程也可能造成部分脆弱细胞亚群丢失。因此，恶性B细胞的判定通常需结合CCND1表达、轻链限制、拷贝数变异（CNV）推断及B细胞受体（BCR）克隆信息进行综合判断，以提高注释可靠性。

2. 单细胞B/T细胞受体测序（scBCR/TCR-seq）：scBCR/TCR-seq能在单细胞层面进行受体序列与转录状态分析，能判断克隆身份并追踪克隆谱系[10]。在MCL中，scBCR-seq常与scRNA-seq联合使用，用于确认恶性克隆属性、比较优势克隆与亚克隆之间的差异，并在纵向分析中观察复发相关克隆的扩增和演变[9],[11]。scTCR-seq则可用于评估T细胞克隆扩增与功能状态之间的关系，为理解包括MCL在内的B细胞淋巴瘤免疫微环境中T细胞失能、耗竭及应答异质性提供线索。

3. 单细胞DNA测序（scDNA-seq）、单细胞表观遗传学与多模态整合：scDNA-seq适用于解析突变共存关系、拷贝数异常和克隆结构，在MCL复发演化和耐药相关克隆追踪中具有潜在价值[11]。单细胞染色质可及性测序（scATAC-seq）可从染色质开放状态推断关键转录因子活性及其调控网络，有望为明确MCL异常转录调控机制提供补充信息[12]。此外，多模态技术则通过在同一细胞内同时获取多个组学层面的信息进一步提升了单细胞注释与机制解释的深度。常见形式包括蛋白与转录联合测定，如CITE-seq（cellular indexing of transcriptomes and epitopes by sequencing），可在同一细胞内同步获取表面蛋白和转录信息，从而减少仅依赖转录本注释所带来的偏差[13]；转录与染色质联合分析则有助于将状态差异与其上游调控程序相连接。目前研究对象仅为MCL的多模态研究仍相对有限，但Roider等[14]将CITE-seq、scTCR-seq及多重免疫荧光（mIF）整合，构建了包括MCL在内的结内B细胞淋巴瘤T细胞参考图谱，为MCL T细胞亚群分层与空间验证提供了更可靠的参照。值得注意的是，新型功能导向的多模态单细胞策略正逐步突破传统转录组分析的局限。Zhang等[15]将单细胞生物物理特征与转录组信息关联分析，发现浮力质量和细胞刚度不仅可提示MCL细胞状态，还与B淋巴细胞酪氨酸激酶（BLK）、分化簇79A（CD79A）等BCR相关基因表达及BTK抑制剂敏感性相关，提示生物物理与转录组整合有望为MCL功能异质性与药物反应评估补充维度信息。

4. 空间转录组与多重成像技术：常规单细胞测序在组织解离过程中会丢失细胞间的空间邻近信息。作为单细胞组学在空间维度的重要补充与延伸，空间转录组学与多重成像技术，如mIF、组织成像质谱流式细胞术（IMC）等，可在保留原有组织结构的基础上，获得基因或蛋白表达与空间定位特征[16]。在MCL的研究中，这类技术不仅可描述肿瘤细胞密度较高区域与肿瘤细胞相对稀少区域的免疫细胞组成差异，还可识别与免疫抑制相关细胞的特征，并推测潜在细胞互作，为候选机制提供原位层面的验证[14],[17]–[18]。需要注意的是，空间转录组通常需要在通量与空间分辨率之间进行权衡，而mIF及IMC虽具备较强的原位验证能力，但检测标记数量和分析流程仍受限制；因此，空间技术更适合作为单细胞发现的定位验证与机制解析的有力补充，而非完全取代解离式单细胞测序。

二、单细胞组学揭示的MCL主要生物学特征

单细胞组学及相关空间与多组学技术的发展，使研究者得以从细胞层面描绘MCL的细胞状态、克隆结构及其与微环境的相互作用。现有证据总体提示，MCL的关键生物学问题可以归纳为以下3点：①诊断时已存在的亚克隆和细胞状态异质性；②随疾病进展和治疗压力发生的微环境重塑；③由肿瘤内在程序与外在免疫生态共同驱动的耐药演进。因此，将分别从肿瘤细胞异质性、微环境生态及治疗耐药3个层面进行归纳。

1. 肿瘤内异质性与克隆演化：单细胞研究进一步表明，MCL并非由单一同质性恶性克隆构成，而是在诊断阶段便存在可检测的细胞状态差异和亚克隆结构。早期scRNA-seq研究在多药耐药MCL中已识别出多个恶性B细胞亚群，其转录程序分别富集于免疫逃逸、药物代谢、DNA损伤修复及抗凋亡等相关通路[8]。Zhang等[7]对伊布替尼敏感与耐药患者的纵向样本进行单细胞分析，发现耐药病例中存在时间依赖性的肿瘤演化，部分耐药亚克隆获得17号染色体长臂增益，并伴抗凋亡能力增强，其中编码生存素Survivin的位于17q的杆状病毒凋亡抑制蛋白重复序列5（BIRC5）基因表达上调，与疾病进展相一致。以上结果提示MCL耐药并非治疗后发生的新事件，而是建立在诊断时已存在的低频异质性克隆的基础之上。

Wan等[9]进一步整合scRNA-seq、scBCR-seq与全基因组测序（WGS）对原发及复发MCL进行分析，结果显示MCL在诊断时即存在可检出的肿瘤内异质性；在疾病发展过程中，患者的演化路径存在差异，可表现为少数克隆在治疗压力下扩增，也可获得新的突变和拷贝数异常，或迁移到不同的微环境生态位。Manakov等[19]关于首次复发配对的研究进一步提示，具有治疗抵抗特征的亚克隆可在诊断时以低频形式存在，并可能伴随与解剖部位相关的演化。以上结果提示，MCL复发并不是沿着同一路径发生，而是肿瘤本身的异质性与治疗选择压力共同构成了复发演进的共同基础，发展轨迹具有明显的个体化特点。

在方法学层面，不同单细胞平台揭示的是MCL克隆演化与耐药形成的不同切面。scRNA-seq更适于描绘细胞状态与通路活性，scBCR-seq有助于确认克隆身份并追踪谱系，而scDNA-seq则更适于解析突变共存关系和克隆结构。Thompson等[11]在接受BTKi和BCL2抑制剂治疗的CLL或MCL患者中观察到，单细胞DNA层面的耐药图谱高度复杂，多个耐药相关遗传事件可在不同亚克隆中并行。因此，对MCL克隆演化的理解不能仅停留在“是否出现新突变”，还应同时关注遗传事件、转录状态和生态位迁移之间的耦合关系。

除遗传学差异外，MCL还存在与侵袭性表型相关的转录重编程现象。Valentin Hansen等[20]的单细胞转录组研究结果显示部分性别决定区Y相关高迁移率族盒转录因子（SOX）11阳性MCL细胞存在SOX4表达，在母细胞样亚型中更为明显，提示SOX4相关程序可能与部分患者的侵袭性转录特征有关。综合现有研究，MCL异质性不仅与存在的基因突变负荷以及突变类型相关，还与不同遗传背景下恶性B细胞不同的增殖、应激、免疫逃逸或存活状态相关，这些状态差异本身就可能决定后续治疗反应和复发。

2. 肿瘤微环境重塑与免疫逃逸：MCL的临床异质性不仅来源于肿瘤细胞本身，也与其所处的微环境相关。单细胞及相关空间组学研究提示，MCL常呈现免疫效应功能受损而抑制性信号通路占主导的微环境特征[8]–[9],[14],[17],[21]。相关机制与空间研究进一步表明，不同遗传背景下MCL可呈现不同的免疫生态模式[18],[22]。

T细胞功能紊乱是目前证据最为充分的MCL免疫微环境特征之一。Jiang等[21]对CAR-T细胞治疗后复发的MCL患者样本进行单细胞分析，发现复发后T细胞尤其是细胞毒性T细胞比例下降、髓系细胞增加，耗竭T细胞以含免疫球蛋白和免疫受体酪氨酸抑制基序结构域的T细胞免疫受体（TIGIT）、淋巴细胞活化基因3蛋白（LAG3）和CD96等检查点分子表达为特征，其中TIGIT的上调最为突出；体外实验验证，联合阻断TIGIT与程序性死亡受体1（PD-1）可部分恢复效应T细胞功能，提示TIGIT相关抑制网络可能是CAR-T细胞治疗后复发/耐药的关键机制之一[21]。Roider等[14]构建的结内B细胞淋巴瘤T细胞图谱提示，包括MCL在内的多种淋巴瘤在T细胞亚群构成和空间分布上存在明显差异，支持MCL的T细胞失衡并非单一耗竭模式。Lokhande等[17]的空间组学研究进一步发现，终末分化阶段的CD57阳性T细胞在MCL中相对富集，并与TIGIT、程序性死亡配体1（PD-L1）、LAG3等抑制分子的高表达相关。

除T细胞外，相关机制与空间研究还提示髓系细胞和调节性T细胞（Treg）也参与构建免疫抑制性生态位。Balsas等[22]证实，SOX11通过促进CD70表达，参与形成Treg富集的微环境，与侵袭性MCL表型相关。Wan等[9]在原发与复发MCL的配对样本中观察到，疾病进展阶段恶性细胞与微环境细胞之间的相互作用增强，并在复发组织中检测到较高的CD70-CD27相关信号，提示免疫抑制性作用可能参与MCL进展与复发。此外，Sharma等[18]结合转录组与IMC研究发现，TP53异常MCL常表现出免疫浸润但功能受损的特征，并伴M2样髓系细胞富集，而ATM突变相关病例则更偏向免疫学上的“冷”肿瘤，提示不同遗传背景下的微环境模式不同。

3. 单细胞视角下的治疗耐药机制：结合前述单细胞研究结果，MCL耐药大致可归为3个相互关联的层面：①克隆演化层面：预存的低频高危或耐药相关亚克隆可在治疗压力下被选择性扩增，并可呈现亚克隆并行演化[7],[9],[11],[19]。针对pirtobrutinib（非共价BTKi）的单细胞多组学研究进一步将耐药分析扩展至scRNA-seq与scATAC-seq等整合层面，提示pirtobrutinib耐药可能同时涉及遗传性变异及表观遗传或转录组重编程[23]。②肿瘤内在程序层面：恶性B细胞通过状态重编程维持存活与信号依赖，例如SOX11相关程序可通过配对盒基因5（PAX5）/CD19轴参与BTKi耐药表型维持，而TP53功能受损可通过解除蛋白酪氨酸磷酸酶非受体型6（PTPN6，其编码蛋白又称含SH2结构域蛋白酪氨酸磷酸酶1，即SHP-1）对BCR信号的抑制而驱动高危表型转化[18],[24]。③微环境层面：免疫抑制性T细胞、Treg和髓系细胞可共同构成治疗保护性生态位，使肿瘤在外源治疗压力下持续存活[9],[21]–[22],[25]。

在这一框架下，除了前述的TIGIT相关T细胞耗竭网络外[21]，现有研究进一步揭示了几条具有代表性的耐药相关通路。Yun等[25]发现，单核/巨噬细胞来源的白细胞介素-1受体拮抗剂（IL-1ra）可直接削弱抗CD19 CAR-T细胞功能，提示髓系细胞并非被动伴随者，而是CAR-T细胞治疗耐药的重要参与者；SOX11-CD70-Treg轴则提示肿瘤细胞可主动塑造抑制性微环境，并可能为治疗抵抗提供生态位基础[22]；在序贯治疗耐药方面，Yan等[26]发现MYC相关信号通路在耐药演进过程中逐步增强，热休克蛋白90（HSP90）-MYC-细胞周期蛋白依赖性激酶9（CDK9）信号轴可能是介导连续性耐药的关键分子机制；后续临床前研究进一步显示，CDK9抑制剂enitociclib在BTKi和CAR-T细胞治疗后耐药的MCL模型中显示出抗肿瘤活性[26]–[27]。综上，MCL耐药并非单一靶点失效所致，而是克隆选择、状态重编程与微环境保护共同作用的结果。需要指出的是，目前上述机制多来自单中心、小样本或特定治疗场景下的研究，其普遍性及临床可转化性仍需在更大样本量的队列和前瞻性研究中进一步验证。

三、单细胞组学在MCL临床转化中的应用前景

1. 风险分层的精细化：目前MCL风险评估仍主要建立在简化MCL国际预后指数（MIPI/MIPI-c）、细胞增殖标志蛋白Ki-67、母细胞样/多形性形态以及TP53异常等临床病理指标基础上[4],[28]–[30]。这些指标具有较好的临床实用性，但反映MCL肿瘤细胞状态和免疫微环境差异仍有限。单细胞研究显示，恶性细胞状态、T细胞耗竭水平、髓系抑制特征及特定配体-受体互作模式等，均可能提供临床指标之外的补充分层信息[9],[14],[17]–[18],[21]。从临床可行性看，现实途径并非是在常规诊疗中普遍开展单细胞测序，而是从单细胞研究结果中筛选出可推广的替代指标。基于当前单细胞研究结果，未来可探索将肿瘤内在特征与免疫生态特征联合纳入的候选组合，例如将TP53/ATM状态、SOX11、Ki-67及T细胞抑制和髓系浸润标志物联合，用于补充现有风险分层；但需注意，这类组合目前仍在探索阶段，尚未形成临床验证标准。此外，Zhang等[15]将单细胞生物物理特征与转录组同步测量，发现浮力质量等生物物理参数与B细胞发育状态及BCR信号通路基因表达相关，且与BTKi的敏感性相关，提示单细胞层面的此类指标可作为未来风险评估和药物反应研究的补充方向。

2. 可检测残留病（MRD）监测的功能拓展：MRD是评估淋巴瘤疗效与复发风险的重要指标，主要关注残留肿瘤细胞或肿瘤克隆的负荷。单细胞组学提示，MRD评估除了关注肿瘤细胞残留数量外，还需进一步关注其生物学特征。理论上，单细胞转录组与BCR克隆信息可同时识别残留细胞的克隆来源、增殖活性及耐药相关状态，从而有望识别具有复发潜能的残留细胞群。外周血取样具有微创和可重复的优势，但MCL特异性的外周血单细胞液体活检证据目前仍有限。现阶段，由于样本处理、成本和通量等限制，单细胞MRD更适用于高危患者的探索性研究。近期提出的Live-cell Pick-Seq（LiP-Seq）平台可在超低频背景下识别并回收治疗后持续存活的活性淋巴瘤细胞，其已用于CD19 CAR-T细胞治疗后持续存留的MCL细胞的单细胞转录组分析，提示针对超低频残留细胞的技术创新有望拓展MCL中MRD的研究边界[31]。未来可行方向是首先利用单细胞研究筛选高危残留特征，然后以流式细胞术、靶向二代测序（NGS）、循环肿瘤DNA或简化表达组合的方式进行临床随访。

3. 联合治疗靶点筛选与个体化治疗优化：单细胞研究在MCL个体化治疗中的潜在价值，主要体现在识别不同患者或不同疾病阶段的关键状态特征及可干预通路，从而为精准干预策略提供机制依据。单细胞研究结果提示以免疫逃逸为主导的CAR-T细胞治疗失败可能由TIGIT相关T细胞耗竭、IL-1ra介导的髓系抑制及SOX11-CD70-Treg相关免疫抑制生态位共同导致，其为后续联合微环境调节策略的探索提供了线索[21]–[22],[25]；对于BCR信号调控异常且与TP53/PTPN6相关的高危患者，现有研究提示其可能具有不同于经典BTK依赖状态的生物学基础，为后续联合干预研究提供了依据[18]；此外，HSP90-MYC-CDK9通路的研究则为理解序贯治疗后的连续耐药提供了新的机制切入点[26]–[27]。然而，单细胞研究目前更多用于提出可检验的生物学假说和筛选治疗靶点，现实价值可能体现在辅助患者分层、优化联合治疗策略以及筛选潜在获益人群，未来仍需前瞻性研究进一步验证。

四、当前挑战与未来方向

尽管单细胞组学进一步拓宽了对MCL生物学特征的认识，但其在临床转化仍面临现实限制。首先，现有研究多为小样本、回顾性或单中心队列，且患者所处的疾病阶段、既往治疗和标本来源等方面差异较大，限制了结果的可比性和外推性。其次，单细胞数据本身会受到技术噪声与前处理偏倚的影响，例如组织解离和冻存过程可导致脆弱细胞亚群丢失，scRNA-seq存在转录本丢失和间接推断CNV的局限，而多模态数据又常面临稀疏性高和批次效应显著的问题[32]。第三，跨平台及跨队列的研究整合仍较困难，不同平台、处理流程和研究队列之间存在显著技术差异[33]。第四，空间技术虽提供原位信息，但在空间分辨率、通量和算法解释方面仍存在现实约束，难以单独完成复杂机制归因[16]。最后，单细胞技术的成本、检测周期与分析复杂度等方面仍难与常规临床决策相匹配。

未来可通过以下方面推进其临床转化：开展多中心、标准化的前瞻性研究，积累具有明确临床结局的纵向样本；加强单细胞组学与病理形态学、靶向测序、循环肿瘤DNA、空间组学和功能实验的整合，提高结果的可解释性和稳定性；结合人工智能与多模态整合方法，提升跨组学数据的联合解读能力；将关键细胞状态及微环境特征进一步提炼为临床可应用的简化标记组合（如mIF评分、靶向NGS或表达特征集），从而推动单细胞研究结果向可推广检测策略的转化。

五、结语

单细胞组学的发展为从细胞层面认识MCL提供新的研究路径。现有研究已在肿瘤内异质性、克隆演化、肿瘤微环境重塑及治疗耐药等方面提供了重要证据。综合现有文献，MCL复发耐药的共性规律并非单一路径，而是诊断时即存在的异质性、治疗压力下的状态重编程，以及免疫抑制性微环境三者共同塑造复发与耐药。总之，目前单细胞技术更适合作为机制研究与标志物发现平台，其在风险分层、MRD监测和个体化联合治疗中的潜在价值，值得进一步探索，但相关结论仍需更大样本量、标准化研究设计和前瞻性队列研究加以验证。
